# Predicting Slurry Pressure Balance with a Long Short-Term Memory Recurrent Neural Network in Difficult Ground Condition

**DOI:** 10.1155/2021/6678355

**Published:** 2021-02-20

**Authors:** Qiang Wang, Xiongyao Xie, Hongjie Yu, Michael A Mooney

**Affiliations:** ^1^Department of Geotechnical Engineering, Tongji University, Shanghai 200092, China; ^2^Department of Civil & Environmental Engineering, Colorado School of Mines, Golden CO80401, USA

## Abstract

The safety of tunneling with shield tunnel boring machines largely depends on the tunnel face pressure, which is currently decided by human operators empirically. Face pressure control is vulnerable to human misjudgment and human errors can cause severe consequences, especially in difficult ground conditions. From a practical perspective, it is therefore beneficial to have a model capable of predicting the tunnel face pressure given operation and the changing geology. In this paper, we propose such a model based on deep learning. More specifically, a long short-term memory (LSTM) recurrent neural network is employed for tunnel face pressure prediction. To correlate with PLC data, linear interpolation is employed to transform the borehole geological data into sequential geological data according to the shield machine position. The slurry pressure in the excavation chamber (SPE) is taken as the output in the case study of Nanning Metro, which is confronted with the clogging problem due to the mixed ground of mudstone and round gravel. The LSTM-based SPE prediction model achieved an overall MAPE and RMSE of 3.83% and 10.3 kPa, respectively, in mudstone rich ground conditions. Factors that influence the model, including different kinds and length of input data and comparison with the traditional machine learning-based model, are also discussed.

## 1. Introduction

With the growing demand of urban tunneling, mechanized tunneling has become increasingly popular due to its construction efficiency and low ground disturbance [1, 2]. Compared to earth pressure balance shield (EPB), slurry pressure balance shield (SPB) is preferred when tunneling in the ground with considerable cobbles and gravels, a common mixed-ground condition encountered when tunneling in southwestern China [3, 4].

**Figure 1:**
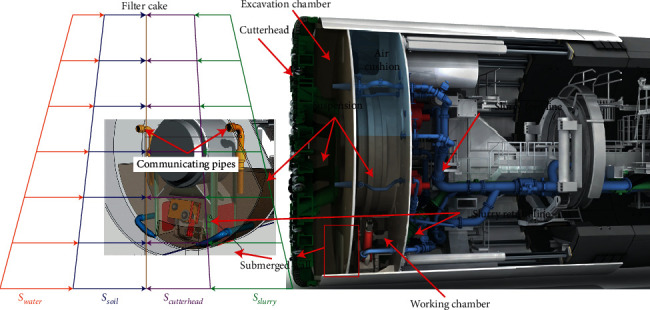
The general arrangement of an SPB shield. The pressure at the tunneling face includes the lateral earth pressure (*S*_*soil*_) and pore water pressure (*S*_*water*_), which is balanced by the cutter head pressure (*S*_*cutterhead*_) and slurry pressure (*S*_*slurry*_) in excavation chamber (modified from Herrenknecht).

Figure 1 shows the typical arrangement of an SPB shield, with the SPB, which has two pressurized chambers: the excavation chamber in the front is filled with bentonite slurry to provide pressure to counterbalance the in-situ pore pressure and lateral earth pressure. Behind the excavation chamber is the working chamber, where the lower portion is filled with slurry and the top portion by air (i.e., air cushion), enabling fine pressure adjustment in the working chamber. Though separated by a submerged wall, the two chambers are hydraulically connected through an opening at the bottom and two communicating pipes at the middle on the submerged wall. During tunneling, the excavated formation soil falls into the excavation chamber and is transported via piping to a slurry treatment plant at the ground surface.

Maintaining proper chamber pressure is critical to the success of SPB tunneling. This is realized manually by operators who decide SPB operations based on experience as well as the reported SPB data (e.g., advance rate, cutterhead torque, and slurry flow rate). With due respect to the value of a seasoned operator, such practice is not ideal when dealing with the difficult ground (e.g., variable geology, mixed face conditions, high clogging potential, and gas-richness), as the relationship of the slurry pressure between the excavation chamber and the working chamber may be ever-changing [3]. Therefore, it would be beneficial to have a model predicting the pressure response and assisting tunneling by suggesting operations in difficult ground conditions. Such a model would also be helpful for the automation driving of the shield machine.

Efforts have been made in this regard, mostly using machine learning- (ML-) based methods [5]. For example, Yeh [6] applied an artificial neural network (ANN) for automatic chamber pressure control in EPB tunneling. In his model, to predict the chamber pressure at next time step (a time step is when the programmable logic controller (PLC) on the tunnel boring machine updates the data measurements, and *t* and *t* + 1 here refer to the current and the next time step), *p* (*t* + 1), both the current and the next time step EPB advance rate, *AR* (*t*) and *AR* (*t* + 1), screw conveyor rotation speed, *ω*s (t) and *ω*s (*t* + 1), and current chamber pressure p(t) are used. After training with 1000 samples, the model is reported to achieve a root mean square error (RMSE) of 13.3 kPa. However, the limited dataset yields high model performance; the author suggested to accumulating additional training examples to improve the prediction accuracy. Similarly, Liu et al. [7] used, instead of ANN, the least square support vector machine with inputs of AR (t), *ω*s (*t*), and *p* (*t*). They proved that their method is capable of predicting earth pressure with RMSE of 8.32 kPa, where there are 400 samples in the training set and 200 samples in the test set. However, they did not address the issue of the influence of geological information. For SPB tunneling, Zhou et al. [8] used the Elman neural network, a variant of ANN, for the prediction of air pressure in the working chamber. Their model takes as inputs *AR*, total thrust force, bentonite suspension level, cutterhead rotation speed, and slurry feed line flow rate at the next time step, as well as the current working chamber air pressure *p*_*air*_ (*t*). The average value per ring in the section of Wuhan Metro Line 2 was adopted to train and test the prediction model while the dataset size is 350 for training and 150 for testing, respectively. The relative error mean value (%) is carried out to evaluate the model performance, which is 0.82% in the training set and 0.55% in the test set. Although a low predicted error was achieved in this model, the feasibility of the model for instantaneous shield tunneling parameters is not addressed. Moreover, the temporal effect is not considered and only one step back's data is taken into account in prediction.

On modern shields, data are recorded by the programmable logic controller (PLC) every 5∼10 seconds (PLC data for short), and the human operators make decisions based on these instantaneous values. The high sampling frequency of the PLC system brings about big data problems. In order to provide better assistance for shield tunneling construction, we harbor the idea that its more reasonable to use big PLC data and geological information to establish the prediction model as well as to consider a longer time effect. None of these works accounted for the contribution of geological conditions and big data problem and only considered the influence of machine operation on pressure. In addition, they all failed to consider the longer term temporal effect of the operation on chamber pressure variation, which may be the reason behind their low performance. For example, the choice of slurry flow rate would cause the change of slurry density in both chambers, whose influence will likely last more than a one-time step forward. However, considering a long temporal effect simply by adding more inputs in the previous time steps to the model is intractable, and a new learning method will have to be used.

In the last decade, some key breakthroughs have made using deep learning to deal with big data problems both in academia and industry [9, 10]. In this paper, we will utilize the deep learning method recurrent neural network (RNN), designed to deal with the time series regression problem and capable of considering all historical information when making new predictions [11]. Specifically, the long short-term memory (LSTM) neural network (NN), a popular variant of RNN [12], will be used. The LSTM-based prediction model has been successfully employed in several cases of time series prediction when considering historical information, such as short wind speed [13], sea surface temperature [14], soil moisture [15], animal behavior pattern [16], traffic speed [17, 18], travel time [19], and rail transit passenger flow [20], showing outstanding results. Recently, Yang et al. [21] presented the LSTM network to predict the periodic landslide displacement, which was found to properly model the dynamic characteristics of landslides than static models and make full use of the historical information. They employed the last 12 data with a sampling interval of one month as input sequence. Liu et al. [22] presented an LSTM-based model for predicting the vibration frequency in the structural health monitoring of machinery or civil structures, which conducted a time step of 6 and a sampling interval of 10^−2^s. Their model yielded a dataset with 225,000,000 simulated signals with a size of 1000 GB, which shows the advantages of LSTM network in dealing with big data problems. Kim et al. [23] proposed spatial partitioning of the hall and an occupancy prediction model based on LSTM to solve the problem of its spatial volume and irregular movements of visitors.

Gao et al. [11] proposed the real-time prediction of tunneling parameters (e.g., the torque, the velocity, the thrust, and chamber pressure) using traditional RNN, LSTM, and gated recurrent unit (GRU) neural networks. The time step of 5 was used in the prediction model but only 3000 samples were presented. Besides, the influence of geological properties was not considered in their research. They did not make clear the effect of input PLC data on the model performance. From the abovementioned applications of the LSTM neural network in the time series prediction problem, we hypothesize it would be beneficial to apply a deep learning-based LSTM network for the tunnel face pressure prediction during SPB excavation.

Our main contributions can be summarized as follows: firstly, we developed the SPE prediction model with multivariable tunneling parameters (not including the SPE) together with the geological parameters at time *t*-1 to *t*-*k* as inputs and the SPE at time *t* as output. Secondly, we investigated the model performance in the different ground conditions, where the clogging problem induced great fluctuations of SPE. To overcome the difficulties of SPE prediction in mudstone rich areas, we developed a deep learning model, which improved the prediction accuracy in the mudstone rich areas. Thirdly, we explore the importance of the input PLC parameters and geological parameters on SPE prediction and using 36% features to achieve a 95% prediction accuracy measured by the *R*^2^ in the proposed model.

The remainder of the paper is organized as follows: in Section 2, we introduce the recurrent neural network and, specifically, the LSTM-based prediction model proposed in this work.  In Section 3, a case study of the Nanning Metro Line 1 is presented for model demonstration. A discussion is presented in Section 4 before presenting the conclusions in Section 5.

## 2. Methodology

### 2.1. RNN, DFN, and LSTM

**Figure 2:**
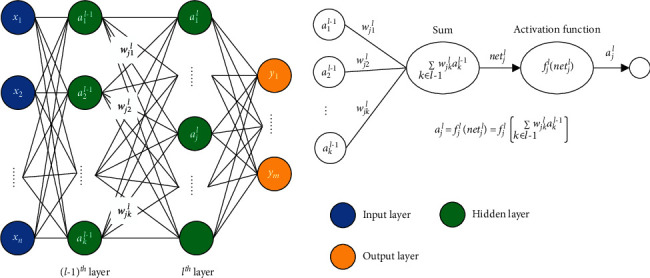
Typical architecture of deep feedforward network with two hidden layers; $\overset{⟶}{x}$ and $\overset{⟶}{y}$ are the inputs and outputs of DFN; *w*_*jk*_^*l*^ is the weight of the connection from the *k*^*th*^ neuron in the (*l−*1)^*th*^ layer to the *j*^*th*^ neuron in the *l*^*th*^ layer*; f* is the activation function.

**Figure 3:**
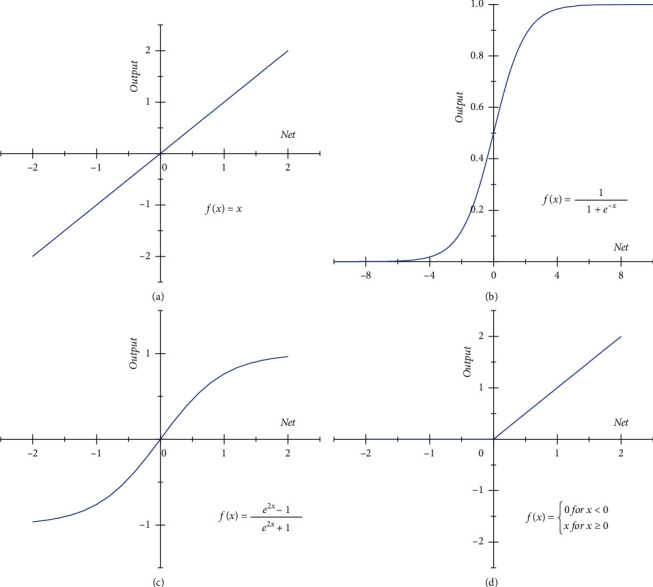
Activation functions in DFN and LSTM network. (a) Linear. (b) Sigmoid. (c) Tanh. (d) ReLU.

Figure 2 illustrates the typical architecture of a deep feedforward network (DFN) and the calculation of the output of the *j*^*th*^ neuron in the *l*^*th*^ layer, *a*_*j*_^*l*^. There are two steps in the calculation of the neuron: summation and activation. Summation relies on the weight matrix that will be learned by the neural network, and activation depends on the choice of the activation function.  Figure 3 shows four kinds of activation functions that are commonly used.

**Figure 4:**
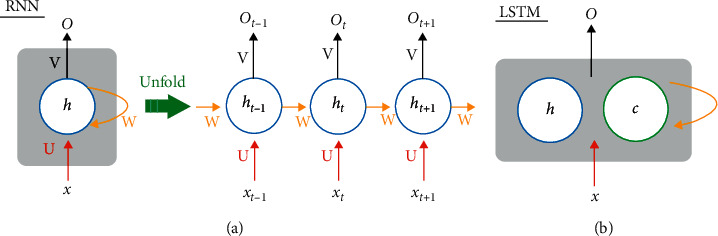
(a) Recurrent neural network and its unfolding in time, where *x, h,* and *O* are, respectively, the input layer, hidden layer, and output layer, while *U*, *W*, and *V* are the corresponding weight matrixes; (b) in contrast, the LSTM's hidden layer has one more component, *c,* the cell state.

Figure 4(a) shows the general structure of an RNN and its unfolding in time. Compared to DFN, the major difference of RNN is the existence of a self-loop in its hidden layer, which allows information in the previous time step to be stored and used. In making predictions, the RNN takes one input (*x*_*t*_) at a time, together with the maintained hidden state (*h*_*t*_) to determine the current output (*o*_*t*_). The behavior of RNN is controlled by its parameters (i.e., matrix *U*, *V*, *W*), which are shared across all time steps and determined during training.

**Figure 5:**
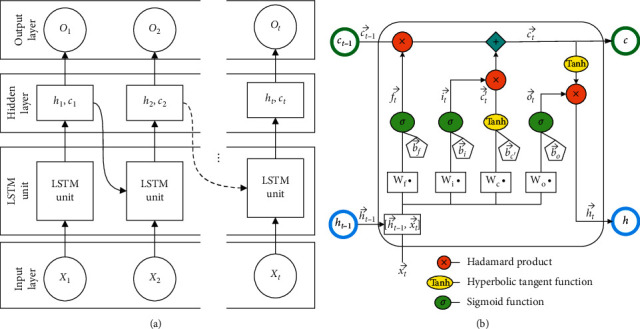
(a) Structure of LSTM with one LSTM layer; the hidden layer contains a special cell state *c*_*t*_ to save the long-term state. (b) Detailed view inside the LSTM layer.

However, both theoretical and empirical evidences suggest that an RNN cannot store information for long and struggles to learn long-term dependency [24, 25]. To this end, an LSTM network with a cell state explicit memory unit (Figure 4(b)) was proposed [12, 26]. The equipped cell state can accumulate past information and has a forget mechanism to control when to erase the past memory. In Figure 5(a), we show the LSTM network's unfolded structure, and the zoom-in view of the LSTM network unit is given in Figure 5(b).

The complicated forward pass calculation of the LSTM network is summarized in equation (1), where ${\overset{⟶}{f}}_{t}$, ${\overset{⟶}{i}}_{t}$, and ${\overset{⟶}{o}}_{t}$ are the values of the forget, input, and output gates, all bounded between 0 and 1. *σ*(·) and tanh(·) are the sigmoid and hyperbolic tangent functions, respectively. ⊙ stands for the element-wise multiplication:

(1) $$\begin{array}{c} {\overset{⟶}{f}}_{t}=\sigma \left(W_{f}\cdot \left[{\overset{⟶}{h}}_{t-1},{\overset{⟶}{x}}_{t}\right]+{\overset{⟶}{b}}_{f}\right),\\ {\overset{⟶}{i}}_{t}=\sigma \left(W_{i}\cdot \left[{\overset{⟶}{h}}_{t-1},{\overset{⟶}{x}}_{t}\right]+{\overset{⟶}{b}}_{i}\right),\\ {\overset{⟶}{o}}_{t}=\sigma \left(W_{o}\cdot \left[{\overset{⟶}{h}}_{t-1},{\overset{⟶}{x}}_{t}\right]+{\overset{⟶}{b}}_{o}\right),\\ {\overset{⟶}{c}}_{t}^{\prime }=\tanh\left(W_{c}\cdot \left[{\overset{⟶}{h}}_{t-1},{\overset{⟶}{x}}_{t}\right]+{\overset{⟶}{b}}_{c^{\prime }}\right),\\ {\overset{⟶}{c}}_{t}={\overset{⟶}{f}}_{t}⊙{\overset{⟶}{c}}_{t-1}+{\overset{⟶}{i}}_{t}⊙{\overset{⟶}{c^{\prime }}}_{t},\\ {\overset{⟶}{h}}_{t}={\overset{⟶}{o}}_{t}⊙\;\tanh\left({\overset{⟶}{c}}_{t}\right). \end{array}$$

The hidden state of the current LSTM network unit ${\overset{⟶}{h}}_{t}$ hinges on both the input ${\overset{⟶}{x}}_{t}$ and the previous hidden state ${\overset{⟶}{h}}_{t-1}$, and is further regulated by $\tanh\left({\overset{⟶}{c}}_{t}\right)$ to capture the network memory, which can be either strong or weak (hence the long- or short-term). ${\overset{⟶}{c}}_{t}$ is the current cell state and is determined from both the previous state and the current inputs.

The training of the LSTM network is the process of determining **W** and *b*, the weight matrix and bias, of the three gates,${\overset{⟶}{f}}_{t}$, ${\overset{⟶}{i}}_{t}$, and ${\overset{⟶}{o}}_{t}$, respectively. In an LSTM network, these weights are fixed across different time steps and the training can be efficiently performed using the “backpropagation through time” algorithm [12].

### 2.2. LSTM-Based Pressure Prediction in SPB Shield Tunneling

**Figure 6:**
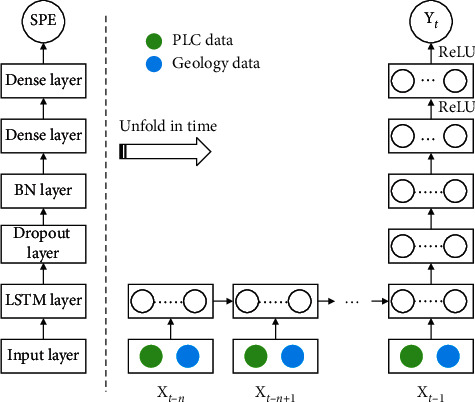
Diagram showing the proposed LSTM model structure (left) and its unfolding in time (right).

The instantaneous slurry pressure in the excavation chamber (SPE) has a significant effect on the tunneling face stability, and its fluctuation is determined by both the machine operation and ground condition. As the TBM shield tunneling process is of high “inertia,” the operation of a limited history should be considered. In Figure 6, we show the structure of the LSTM network model proposed in this paper. Besides the LSTM network layer, some additional layers are also used and will be discussed below.

In the input layer, both the PLC data (i.e., SPB recorded data) and the geology data are included. Such a sequence of input vectors is passed into the LSTM network layer, where the calculation described above is performed. The number of neurons in the LSTM network layer is a hyperparameter of the model, which will be determined via numerical experiments.

The last output of the LSTM network layer is then fed to a dropout layer. The idea of the dropout was first proposed to reduce overfitting risk in training deep neural network [27]. By ignoring some neurons (i.e., set their output to zero) during training at random with some probabilities, the codependency among features can be broken and the network is forced to learn more robustly. When dropout is implemented, only a subportion of the neural network is trained in each epoch; therefore, it acts as a special form of model regularization. As the number of neurons in the LSTM layer, the dropout probability (or ratio) is another hyperparameter to be determined.

Using a batch normalization (BN) layer, the output of the dropout layer in each mini-batch is standardized, yielding them of zero mean and unit variance. Doing so will help to speed up the training and reduce the model's sensitivity to poor network initialization [28].

Following the BN layer are two fully-connected dense layers, implemented to gradually compress the output to a lower dimension for final output. The activation function used in these two dense layers is ReLU, which has the advantage of biological plausibility, better gradient propagation, and efficient computation [29].

The Glorot uniform initialization method [30] and the Nadam optimization method [31] are employed to obtain good generalization performance. Besides, early-stop [32] is used to stop the training process with the monitor parameter of the loss function in the validation set, which is beneficial for preventing the overfitting problem.

## 3. Case Study of Nanning Metro

**Figure 7:**
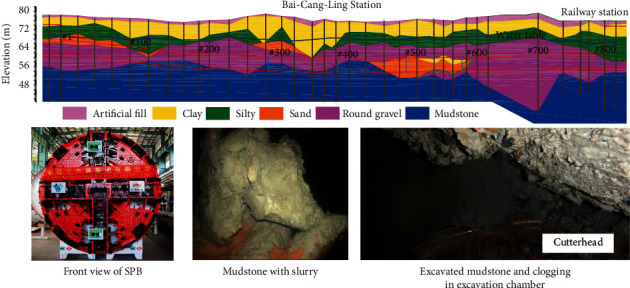
Geological profile of the BR section according to the geotechnical investigation report including the front view of shield machine and clogging situations.

In this study, data gathered from Bai-Cang-Ling Station to the Railway Station (BR section, shown in Figure 7) of Metro Line 1 in Nanning, China, is used [2]. The section consists of 806 rings in total, 1.209 km in length, and is excavated using a Herrenknecht SPB with a diameter of 6.28 m. The excavation was performed from December 2014 to June 2015.

The SPB shield machine was designed for the round gravel condition, which is suitable for settlement control in the urban areas. The ground conditions with round gravel can be regarded as the normal ground condition in this study. However, in the ground conditions with mudstone, as shown in Figure 7, ring #120 to 220, and ring #283 to 470, the SPB shield machine suffered from the problem of clogging, where the tunneling efficiency was much lower than in the normal ground conditions. Moreover, tunnel face passive failure often occurred in the mudstone area, which is harmful to settlement control. Consequently, we take the mudstone area as difficult ground conditions.

### 3.1. Geological Data

A total of 36 boreholes were drilled in the vicinity of the BR section as part of the geological site investigation. These boreholes provide a detailed record of soil types, basic physical properties such as unit weight, porosity, Atterberg limits, moisture content, and particle size distribution, as well as in-situ groundwater table measurements. Using these data, a geological report was prepared for construction reference.

To obtain the geological information at each ring from the sparse borehole (on average 23 rings between boreholes), a linear interpolation is performed. Specifically, all boreholes are projected onto a 2D vertical plane following the centerline of the tunnel alignment, and the geological information at each ring location is interpolated at the center point of the ring based on the instantaneous positions of SPB.

According to the report, the ground in this section mainly consists of round gravel and mudstone, the latter of which is generally located between ring #120 to 220 and ring #283 to 470, as is shown in Figure 7. A particular problem when tunneling with SPB in the clayey ground such as mudstone is clogging [3]. Here, the excavated material may clog the opening of the cutterhead or the submerged wall, obstructing the smooth circulation of slurry. When clogging occurs, it can lead to extreme pressure fluctuation in the excavation chamber and result in increased tool wear and reduced SPB advance rate [3, 33], impacting the safety of excavation and the longevity of the machine. These extreme pressure fluctuations make it difficult for slurry pressure prediction.

### 3.2. PLC Data

To assist machine operation, modern SPBs are often well instrumented to gather data ranging from human operations (e.g., slurry feed/return line flow rate and cutterhead rotation speed) to resulting machine reactions (e.g., cutterhead torque, advance rate, and fluid pressures). The SPB shield data are automatically recorded by a PLC every 10 seconds.

**Figure 8:**
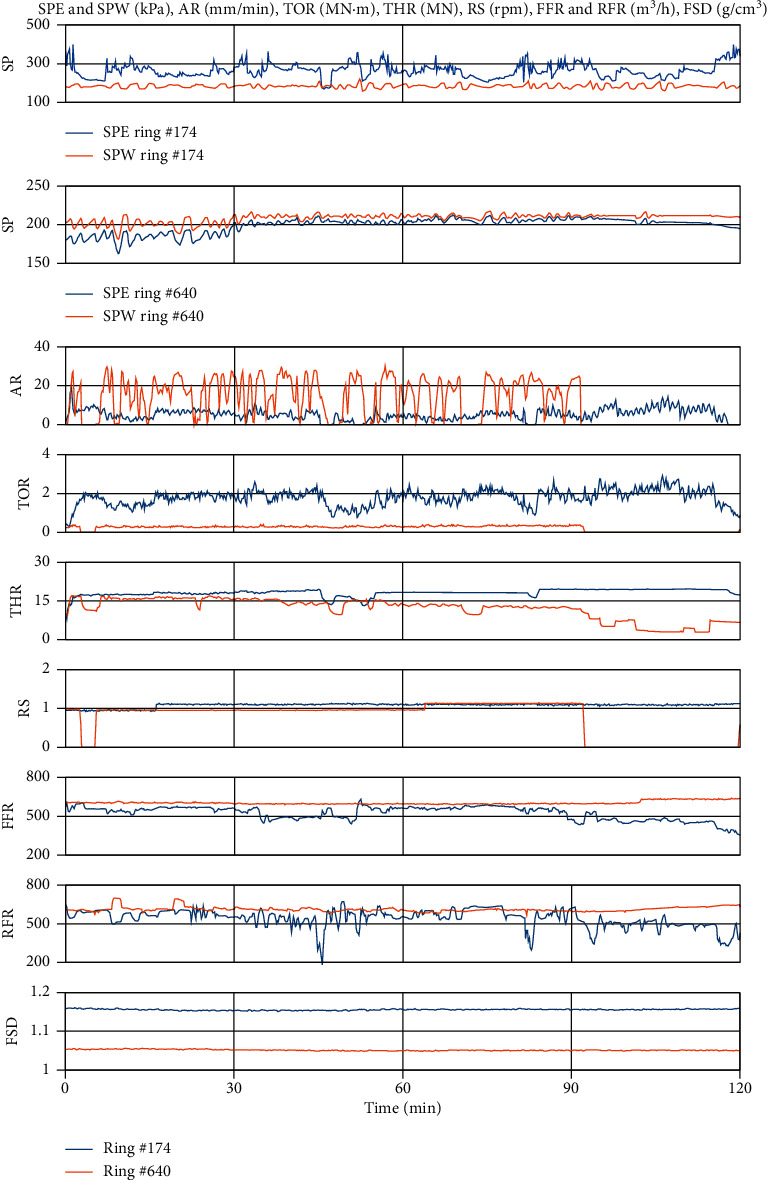
The PLC recorded data on ring #174 and # 640, from top to bottom, are the slurry pressure in the excavation chamber (SPE) and the working chamber (SPW), advance rate (AR), cutterhead torque (TOR), total thrust (THR), cutterhead rotation speed (RS), feedline slurry flow rate (FFR), return line slurry flow rate (RFR), and feedline slurry density (FSD), respectively.

In Figure 8, an example of the recorded data is plotted for ring #174 and #640. The former one represents clogging conditions while the latter one was in normal condition. We plot the two hours of data for each ring, which includes the measured slurry pressures in the excavation chamber (SPE) and working chamber (SPW), SPB advance rate (AR), cutterhead rotation speed (RS) and torque (TOR), and thrust force (THR), as well as slurry flow rate (both the feed (FFR) and return lines (RFR)) and density (only feed line (FSD) as density sensor in return line did not work well at the latter half of BR section). Since ring #321 is in the mudstone ground, clogging is observed [3] and is characterized by an SPE fluctuation as much as 300 kPa, indicating the possible jamming of the submerged wall opening. The clogging results in that the relationship between SPE and SPW is ever-changing in mudstone rich areas, which brings about great difficulties for tunnel face stability control. As a result, significant variation of machine advance rate, cutterhead torque, thrust force, and return line slurry flow rate is observed, which undermines the safety and efficiency of tunneling. Meanwhile, ring #640 locates in the round gravel ground, no clogging occurred, thus all these parameters are in normal condition. More specifically, the SPE and SPW change smoothness, as well as larger AR and smaller cutterhead torque are observed than in ring #174.

**Figure 9:**
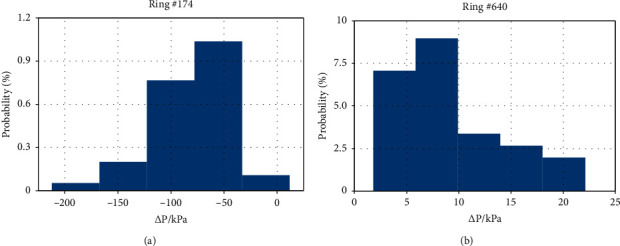
Δ*P* distributions of ring #174 and #640.

The SPE and SPW are measured by pressure sensors located at the spring line of both chambers. We define the differences between SPW and SPE as Δ*P*=SPW − SPE. Normally, Δ*P* is in the range of 0 to 20 kPa (Figure 9(b)), but when clogging occurs, the Δ*P* will be in the range of −50 to −150 kPa (Figure 9(a)).

### 3.3. LSTM Model Implementation

#### 3.3.1. Model Input

There are two types of model inputs. On the SPB side, the PLC has recoded the machine operation and reaction during tunneling and will be used. Specifically, measurements from eight parameters are used, including the slurry pressure in the working chamber (SPW), machine advance rate (AR), cutterhead torque (TOR) and rotation speed (RS), total thrust force (THR), flow rates of the slurry feed line (FFR) and return line (RFR), and slurry density in the feed line (FSD). We employ the PLC data at all tunneling periods, both including the excavation period (AR > 0) and the stoppage period (AR = 0).  Table 1 summarizes the statistics for the PLC input and output parameters.

**Figure 10:**
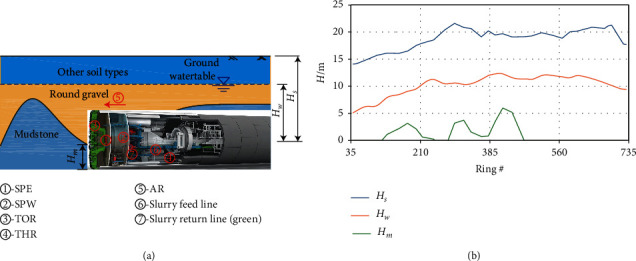
(a) Schematic diagram of SPB in the mixed ground of round gravel and mudstone in the BR section and the input parameters of the prediction model; (b) average values per ring of tunnel buried depth, groundwater table, and mudstone thickness in excavation face.

As for the geology, both the spring line tunnel buried depth *H*_*s*_ and groundwater table *H*_*w*_ interpolated from the borehole data are used. Besides, as the presence of mudstone will severely influence the chamber pressure, the thickness of the, *H*_*m*_, is also included, as shown in equation (2). These soil parameters are chosen due to the calculation of the slurry pressure in SPB tunneling [34]. The definition of these three parameters is given in Figure 10(a) (mudstone formation within the excavation envelope). The average values per ring of geological data are demonstrated in Figure 10(b). We can see that the *H*_*s*_ is about 14 m to 22 m, while the *H*_*w*_ is about 5 m to 12 m, and the *H*_*m*_ ranges from 0 to 6 m.

(2) $$\begin{array}{c} {\text{SPE}}^{t}=f\left({H_{s},H_{w},H_{m},\text{SPW},\text{AR},\text{TOR},\text{RS},\text{THR},\text{FFR},\text{RFR},\text{FSD}|}^{t-1,t-2,\ldots ,t-n}\right). \end{array}$$

The selection of model input parameters is determined by tunneling domain knowledge, and a further discussion on their relative importance is given in Section 4.2.

#### 3.3.2. Data Preprocessing

Data cleaning work is conducted by removing the outliers of the tunneling data according to the measurement range of different sensors. For example, the maximum value of SPE is designed as 500 kPa, so the SPE measured by the PLC system at time *t* is larger than 500 kPa; all the tunneling data at time *t* will be removed.

After the removal of abnormal data, data of 665 rings are available, yielding over 1.48 million samples in total. To remove the potential influence of various input scales, all inputs are first normalized between 0 and 1, following

(3) $$\begin{array}{c} X_{\text{norm}}=\frac{X-X_{\min}}{X_{\max}-X_{\min}}. \end{array}$$

Before training, all data are segmented into sequences so that they can be readily fed into the input layer. The sequence length is another hyperparameter and it should neither be too long, as this is burdensome computationally, nor too short, as it limits the temporal dependency the model could possibly discover. In our model, each segment consists of 18 consecutive measurements (i.e., three minutes), which will be discussed in Section 4.1.

After segmentation, in total, 1,487,705 sequences (about 172 days data) are present, which are further randomly split into three sets for training, validation, and testing by a training ratio *η*, *η* ∈ [0.1,0.9], each accounting for *η*, (1 − *η*)/2, and (1 − *η*)/2 of the whole dataset, respectively. The training of the LSTM neural network is conducted with the help of Keras, a high-level neural network API, written in Python capable of running on top of Tensor Flow, CNTK, or Theano [35]. Four Nvidia GeForce GTX 1080 Ti graphics cards are used in the hardware platform.

#### 3.3.3. Hyperparameter Tuning

The proposed model has four hyperparameters, including the number of neurons in the LSTM layer, the batch size, the dropout ratio, and the training ratio. They are used to tradeoff model's empirical performance with generalization ability and should be set properly. Hyperparameters are determined using numerical experiments.

**Figure 11:**
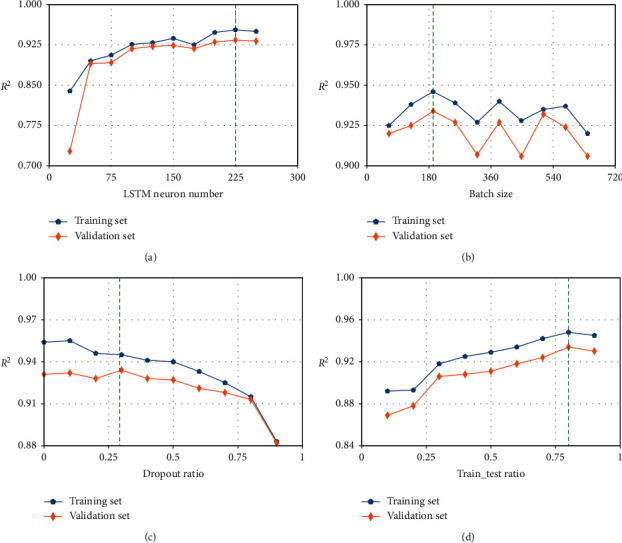
Hyperparameters including neuron number in the LSTM layer, batch size, dropout ratio, and time sequence length tuning process considering the *R*^*2*^.

Due to the large data size, the optimal hyperparameters are determined in a stage-wise fashion: the optimal number of neurons, batch size, drop-out ratio, and training ratio are searched in sequence, based on the model performance on the training and validation set. Two performance metrics are used for model evaluation: the root mean square error (RMSE) and the adjusted coefficient of determination (*R*^2^), which are calculated as

(4) $$\begin{array}{c} \text{RMSE}=\sqrt{\frac{1}{N}{\sum_{i=1}^{N}\left(Y_{i}-{\hat{Y}}_{i}\right)}^{2}}, \end{array}$$

(5) $$\begin{array}{c} \left\{\begin{array}{c} R_{0}^{2}=1-\frac{\sum_{i=1}^{N}{\left(Y_{i}-{\hat{Y}}_{i}\right)}^{2}}{\sum_{i=1}^{N}{\left(Y_{i}-\bar{Y}\right)}^{2}},\\ R^{2}=1-\frac{\left(1-R_{0}^{2}\right)\left(N-1\right)}{\left(N-p-1\right)}, \end{array}\right. \end{array}$$

where *Y*_*i*_ is the measured value, ${\hat{Y}}_{i}$ is the model prediction, *N* is the sample size, and *p* is the input feature number. In Figure 11, the model performances on the training and validation set are plotted. After hyperparameter tuning, the number of neurons in the LSTM of 225, the batch size of 216, and the dropout ratio of 0.3, and the training ratio of 0.8 are selected in our proposed model.

**Figure 12:**
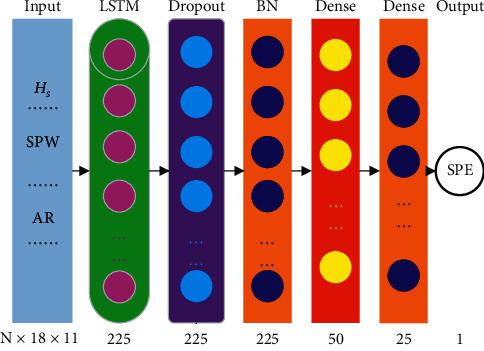
Proposed prediction model structure; N × 18 × 11 means the dimensions of the input layer, where N is the sample number, 18 represents the time step (3 minutes), and 11 is the kinds of the input parameters. Integers such as 225 and 50 are the nodes in each layer, BN is the batch normalization layer, and Dense is the fully-connected layer.

After the hyperparameter tuning, the proposed prediction model structure is shown in Figure 12.

**Figure 13:**
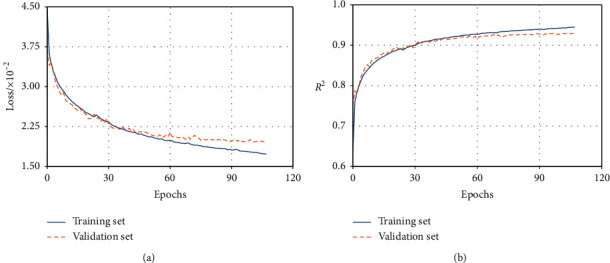
Variations of (a) the loss function and (b) the adjusted coefficient of determination (*R*^*2*^) in the training and validation set.

Figure 13(a) demonstrates the variations of loss function values on the training set and validation set during the training process of the proposed model. We can see that both the training and validation loss decrease as the training epochs increase and the difference between training and validation loss is very small, which indicates the prediction model gains good generalization performance. At the same time, the *R*^2^ increases as the training epochs increase (Figure 13(b)). For 30 epochs, the *R*^2^ value is approximately 0.9, and for 113 epochs, it reaches 0.95 on the training set. Finally, the model achieves an *R*^2^ value of 0.93 on the test set, which is slightly smaller than the value on the training set of 0.95 and similar to the validation set of 0.93.

### 3.4. Results

**Figure 14:**
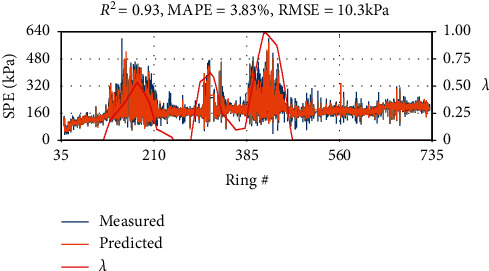
Model prediction performance in the BR section. Measured and predicted SPE with the mudstone distribution along the tunnel alignment in the BR section.

In Figure 14, we plot the model predicted SPE against the measured SPE, along the BR section, together with the *H*_*m*_ distributions. For a better analysis of model performance, the mean absolute percentage error (MAPE) is presented as calculated in equation (6). We also defined the mixed ground ratio*λ* [3] as the ratio of *H*_*m*_ and cutterhead diameter *D* to represent the impact of mudstone, as illustrated in equation (7). The overall RMSE and MAPE are calculated to be 10.3 kPa and 3.83%, respectively, and the adjusted coefficient of determination is found to be *R*^2^ = 0.93 in the test set, suggesting LSTM could model the evolution of SPE with reasonable accuracy.

(6) $$\begin{array}{c} \text{MAPE}=\frac{1}{N}\sum_{i=1}^{N}\left|\frac{Y_{i}-{\hat{Y}}_{i}}{Y_{i}}\right|\times 100, \end{array}$$

(7) $$\begin{array}{c} \lambda =\frac{H_{m}}{D}. \end{array}$$

**Figure 15:**
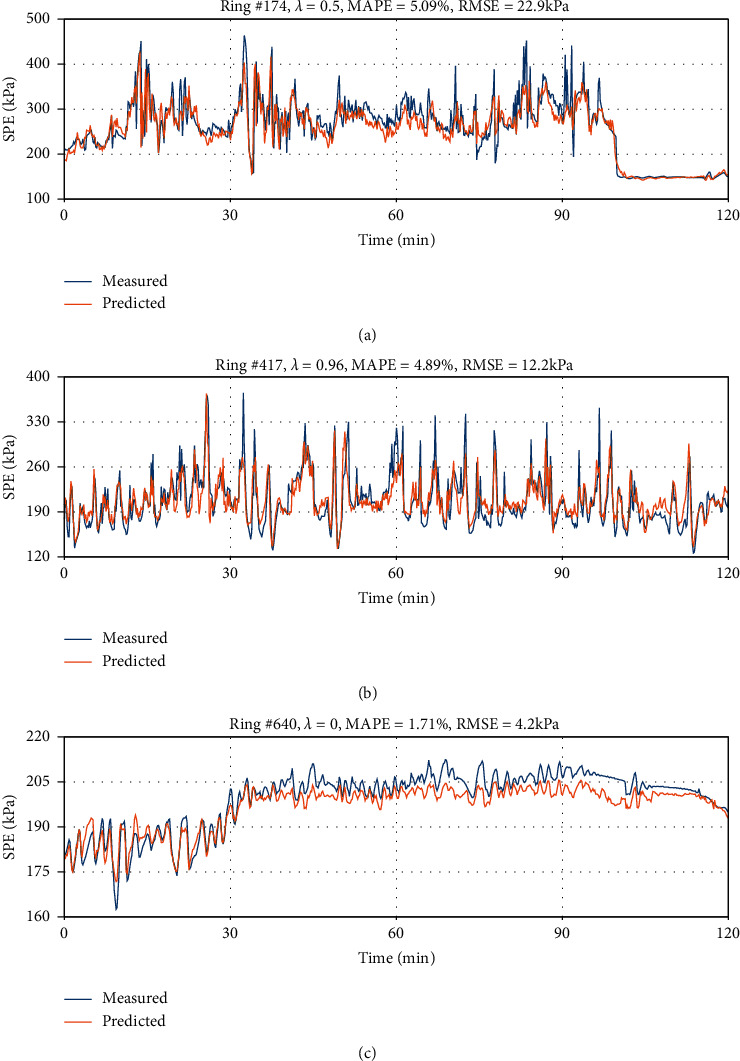
Three typical rings in BR section of the measured and predicted SPE; (a) ring #174 with half mudstone in tunnel face; (b) ring #417 with almost full mudstone in tunnel face; and (c) ring #640 with no mudstone in tunnel face.

In order to investigate the model performance in different ground conditions, we plot three typical rings with different *λ*, as shown in Figures 15(a)–15(c). For ring #174 with *λ*=0.5 (Figure 15(a)), it is observed that the model can well capture the variation of SPE in most cases, only missing some extreme fluctuations. These differences have led to MAPE of 5.09% and RMSE of 22.9 *kPa*, which is larger than the overall dataset MAPE and RMSE. In the ring #417 with *λ*=0.96 (Figure 15(b)), the model performs a little better than in ring #174, which yields a MAPE of 4.89% and an RMSE of 12.2 kPa, but still larger than those on the overall dataset. In the ring #640 with *λ*=0 (Figure 15(c)), the model performs much better than in ring #174 and #417 with a MAPE of 1.71% and an RMSE of 4.2 kPa. We also find differences between the measured and predicted SPE in Figure 15(c), but very small, thus the MAPE and RMSE are much smaller than those in the overall dataset.

**Figure 16:**
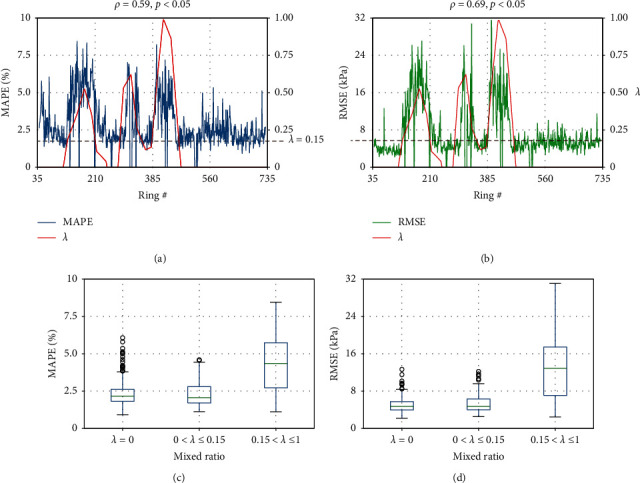
Model performance with the mudstone distribution; (a) MAPE variation per ring with *λ* and (b) RMSE variation per ring with.*λ*

From these three typical rings, we believe that the model performance is related to the mudstone distribution; therefore, we plot the MAPE and RMSE per ring along with the mudstone distribution in Figures 16(a) and 16(b). It can be seen that the large values of MAPE and RMSE are obtained when *λ* > 0.15[3] in most cases. The correlation coefficient *ρ*_MAPE,*λ*_ between MAPE and *λ* is 0.59 and *ρ*_RMSE,*λ*_ between RMSE and *λ* is 0.69, which indicates a strong relationship between the model performance and mudstone distribution. In Figures 16(c) and 16(d), we employ boxplot to show the spread and centers of MAPE and RMSE with different ranges of *λ*. As previous research [3] suggested, when *λ* > 0.15, clogging is easy to take place. Here, we divided the *λ* into three groups, *λ*=0, *λ* ∈ (0,0.15], and *λ* ∈ (0.15,1], to investigate the distributions of MAPE and RMSE. It can be found that when *λ*=0 and *λ* ∈ (0,0.15], the MAPE and RMSE have similar median values and quartiles, that is, the model performance has no differences when 0 ≤ *λ* ≤ 0.15. However, when 0.15 ≤ *λ* ≤ 1, the model performance becomes a litter worse. The reason about the model performance change in different ground condition may be the much larger fluctuations of SPE than the input parameters. When tunneling in mudstone dominated ground, the pressure is characterized by higher magnitude (up to 500 kPa) and variation. The LSTM-based deep learning model can capture the variation trend according to the input parameter fluctuations but fail to predict the extreme value of measured SPE. The prediction error of extreme values of measured SPE has contributed to large MAPE and RMSE.

As mentioned before, we use the tunneling data both in the excavation period and the stoppage period. Here, we explore the model performance in different construction periods, as listed in Table 2. Very long stoppage time was encountered in the BR section due to the Spring Festival holiday and clogging. When clogging occurred, the operators had to stop the shield machine and tried some other measures to eliminate clogging. Increasing the slurry cycle time to remove the jammed mudstone was frequently conducted. There is a little difference in the model performance in the excavation period and stoppage period. When the shield machine stopped, the SPW may be a good predictor of SPE, thus smaller MAPE and RMSE are obtained than that in the whole dataset.

## 4. Discussion

### 4.1. Comparison with RF, DFN, and SVR

**Figure 17:**
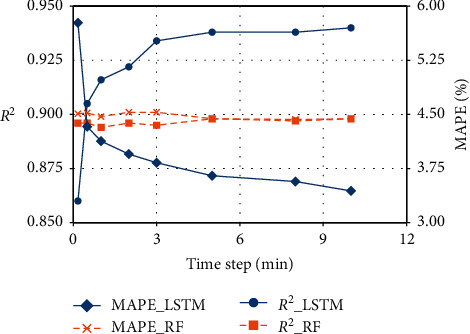
Comparisons between the LSTM model and RF model with a different time step.

To evaluate the LSTM-based prediction model performance, we employ three predictive models including the RF model [36], the deep feedforward network (DFN [37]) model, and the SVR model [38] which are for the SPE prediction here. At first, we will compare the model performance of RF and LSTM in considering time effect, and then we will compare the model performance between the LSTM network and other models. The RF, DFN, and SVR models employ the same training, validation, and test dataset as the proposed LSTM model. The input of these three models will be (*N*, time steps × *n*_features) while the input of the LSTM model is (*N*, time steps,*n*_features). The DFN model structure is similar to the LSTM model, whose hypermeters are determined by the numerical experiments. The hyperparameters of the RF and SVR models are obtained via a randomized search and 3-fold cross-validation [39].  Figure 17 shows the *R*^2^ and MAPE of the LSTM model and RF model with different time steps. It can be told that with the time step increase, the LSTM model performance becomes better while the RF model performance changes very little. Though the RF model has a larger *R*^2^ and a smaller MAPE with a time step of one, the LSTM model can achieve an *R*^2^ value of 0.934 when the time step is 18 (three-minute series). When considering a long time effect, the *R*^2^ value of the LSTM model can be a litter larger and the MAPE of the LSTM model becomes smaller, but longer time effect means more computing resources. Therefore, we conduct a time step of three minutes in the proposed LSTM prediction model. With the comparisons between these two models with different time steps, we can see that the LSTM model can learn more information when considering a longer temporal effect due to its recurrent structure and gating mechanisms.

Table 3 shows *R*^2^ values in the test set and the overall MAPE. The proposed LSTM-based SPE model shows the best performances in both *R*^2^ and MAPE values. The RF model performs a little worse than the proposed LSTM-based model. As a kind of deep learning model, the DFN model performance is worse than the LSTM model because the DFN model cannot consider the time effect. The SVR model achieves the lowest *R*^2^ value and the highest MAPE value, which is unsuitable for SPE prediction with great fluctuations in the difficult ground conditions. Also, we consider the stacked LSTM network structure, mentioned in [40], but the overfitting problem limits the applications of stacked LSTM network in the SPE prediction with great fluctuations in the difficult ground conditions.

### 4.2. Feature Importance

**Figure 18:**
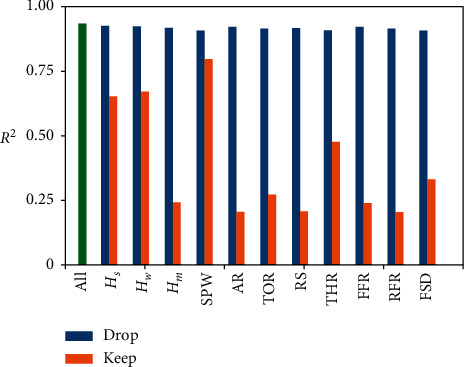
*R* ^*2*^ value comparison considering single input features; drop means drop this feature, keep means only keep this feature, and all means the proposed model.

In this study, a total of 11 features are selected as the model input, as shown in equation (2). To evaluate the importance of each feature, we first proposed 22 scenarios by dropping or keeping one certain type of input feature and compared the *R*^*2*^ values in the test set with the proposed model, as shown in Figure 18.

By comparing the *R*^*2*^ with the proposed model in Section 3.3, it can be found that by dropping only one feature, there is a little diminution of *R*^*2*^. Among dropping one feature scenarios, SPW, THR, and FSD are the most significant ones with a decrement of *R*^*2*^ about 0.03. When we only use one feature to predict SPE, the model performs poorly in most cases with an *R*^*2*^ value of around 0.25. However, the SPW, *H*_*s*_, and *H*_*w*_ can achieve an *R*^*2*^ value larger than 0.6. Besides, the THR also has an *R*^*2*^ value of around 0.5. The abovementioned rules can be explained by field experience during the SPB shield tunneling. Based on the theoretical calculation of tunnel face stability, the *H*_*s*_ and *H*_*w*_ are the main reasons for soil pressure and water pressure. Meanwhile, the SPW and the total thrust of cutterhead are related to *R*^*2*^ from the perspective of the mechanical equilibrium of SPB; therefore, these four kinds of input features have the greatest impact on the prediction performance of SPE. Although the model performance in different rings has a strong relationship with the mudstone distribution, the *H*_*m*_ has little impact on model performance as *H*_*m*_=0 in the majority of rings.

Based on the single feature importance on model performance, we design additional scenarios to investigate whether we can use fewer features to obtain a good prediction model, as shown in Table 4. We define *δ*=*R*_*i*_^2^/*R*_0_^2^ × 100 as a measure of the different input scenario performances, where *R*_*i*_^2^ is the adjusted coefficient of determination, considering the *i*th input scenarios, and *R*_0_^2^=0.934 is obtained by the proposed model. As shown in Figure 18, the SPW seems a good predictor for SPE, and only using SPW can achieve 85% performance compared to our proposed model. However, the relationship between SPE and SPW is ever-changing in mudstone-rich areas, as illustrated in Figure 8. Therefore, we believe it is better to employ more features as model input to obtain good performance in difficult ground conditions. We first use two kinds of geological data, *H*_*s*_ and *H*_*w*_, and find that the *R*^*2*^ is 0.712 and *δ* is 76%, which means if we just take the buried depth and underground water table as input to predict the SPE, the LSTM model can achieve 76% performance of our proposed model. Then, we add SPW to the input and obtain a fairly good result with *R*^*2*^ of 0.848, which reaches 91% performance compared to our proposed model. Thirdly, only two kinds of PLC data are employed, the SPW and THR. In the third scenario, an *R*^*2*^ of 0.865 is obtained, which is a little better than the second scenario. Finally, we put the four significant features into the model and acquire an *R*^*2*^ of 0.884. That is, we use 36% features and obtain a 95% performance of the proposed model. Identifying significant features that have affected prediction performance is crucial in that it provides insight into how a model may be improved and supports understanding of the shield tunneling process being modeled. It is also important in terms of input feature selection because it can reduce measurement and storage requirements.

## 5. Conclusion

In this paper, a deep learning-based slurry pressure prediction model for SPB has been established using the LSTM network, which predicts the slurry pressure in the excavation chamber with instantaneous tunneling parameters, and the geological data. A case study of the Nanning Metro Tunnel project is included for model demonstration. The conclusions of the paper are as follows:

1. It is suitable for the LSTM network to deal with big data time series prediction problem due to its ability to take the effect of history inputs into account. An SPE prediction model has been put forward, which can achieve a prediction performance of *R*^*2*^ value of 0.934 in Nanning Metro.
2. The overall MAPE and RMSE of SPE in this study are 3.83% and 10.3 *kPa*, respectively. The prediction model performs better in the round gravel ground than in the mudstone ground. The SPE prediction model is capable of capturing the variation trend but ignores some peak values in the high clogging potential ground, especially in the mixed ground of half-mudstone and half-round gravel.
3. The influence of the LSTM-based SPE prediction model is demonstrated. More specifically, the influences of input data have been evaluated, and the results indicate that the mudstone, buried depth, underground water table, SPW, and total thrust of cutterhead have a larger effect on prediction accuracy. By feature selection, we can obtain 95% performance of the proposed model with 36% features. Besides, the time step influences the LSTM-based model performance significantly compared with other ML models (e.g., RF, DFN, and SVR), and the LSTM-based deep learning model can learn more information when considering a longer temporal effect.

Despite the abovementioned achievements, however, some further improvements should be made in the deep learning-based prediction model. On the one hand, it is crucial to adopt more kinds of geological data as the model input, which seems more rewarding in actual shield tunneling construction. On the other hand, the output of this model is the instantaneous SPE, which is indirect in contrast with the settlement that the field engineers mainly concern during shield tunneling construction. Meanwhile, the advance rate and attitude of the shield are also their interest as pursuing tunneling efficiency and quality. Therefore, in the following researches, the tunneling parameter prediction model should consider more geological data, especially the soil or rock properties, and employ more parameters as output to provide better guidance for shield operators. If we can build a cycle of prediction and control with the intelligent methods, the tunneling construction would be smarter.

## Figures and Tables

**Table 1:** Statistics of output and input PLC data.

PLC parameter	Unit	Mean	Max.	Min.	Std.
SPE (output)	kPa	172.09	493.30	0	44.28
SPW (input)	kPa	176.25	489.30	0	28.99
AR (input)	mm/min	4.01	50	0	8.83
TOR (input)	MN·m	0.52	5.44	0	0.93
RS (input)	rpm	0.39	2.07	0	0.55
THR (input)	MN	9.49	27.96	0.02	5.93
FFR (input)	m^3^/h	327.70	1482.73	0	352.73
RFR (input)	m^3^/h	337.51	1428.82	0	365.90
FSD (input)	g/cm^3^	1.12	1.41	0	0.06

**Table 2:** LSTM-based deep learning model performance in the different construction periods.

Construction period	Time ratio (%)	MAPE (%)	RMSE (kPa)
Excavation	27.7	4.05	12.9
Stoppage	72.3	3.74	9.3
Total	100	3.83	10.3

**Table 3:** *R* ^2^ and RMSE values of different predictive models.

Metrics\models	LSTM	RF	DFN	SVR
*R* ^2^ in test set	0.934	0.894	0.852	0.812
Overall MAPE (%)	3.83	4.50	5.24	6.15

**Table 4:** Model performance with significant input features.

*i*	Input scenarios	*R*_*i*_^2^	*δ* (%)
1	SPW	0.797	85
2	*H*_*s*_, *H*_*w*_	0.712	76
3	*H*_*s*_, *H*_*w*_, SPW	0.848	91
4	SPW, THR	0.865	93
5	*H*_*s*_, *H*_*w*_, SPW, THR	0.884	95

## Data Availability

The data used to support the findings of this study are included within the article.
